# Enrichment of Apple–Plum Fruit Mousse with Vitamin D_3_ and Sea Buckthorn Oil Using Pectin-Based Encapsulation: A Study of Physicochemical and Sensory Properties

**DOI:** 10.3390/ijms262311480

**Published:** 2025-11-27

**Authors:** Magdalena Krystyjan, Patrycja Majka, Joanna Sobolewska-Zielińska, Katarzyna Turek, Oskar Michalski, Karen Khachatryan, Gohar Khachatryan

**Affiliations:** 1 Department of Carbohydrates Technology and Cereal Processing, Faculty of Food Technology, University of Agriculture in Krakow, Al. Mickiewicza 21, 31-120 Krakow, Poland; magdalena.krystyjan@urk.edu.pl; 2 Department of Food Analysis and Quality Assessment, Faculty of Food Technology, University of Agriculture in Krakow, Al. Mickiewicza 21, 31-120 Krakow, Poland; patrycja.majka@student.urk.edu.pl (P.M.); joanna.sobolewska-zielinska@urk.edu.pl (J.S.-Z.); 3 Department of Animal Product Processing, Faculty of Food Technology, University of Agriculture in Krakow, Al. Mickiewicza 21, 31-120 Krakow, Poland; katarzyna.turek@urk.edu.pl; 4 Department of Chemistry, Faculty of Food Technology, University of Agriculture in Krakow, Al. Mickiewicza 21, 31-120 Krakow, Poland; oskar.michalski@urk.edu.pl; 5 Laboratory of Nanotechnology and Nanomaterials, Faculty of Food Technology, University of Agriculture in Krakow, Al. Mickiewicza 21, 31-120 Krakow, Poland; karen.khachatryan@urk.edu.pl

**Keywords:** functional food, microencapsulation, pectin, vitamin D, sea buckthorn oil

## Abstract

The growing demand for ‘clean label’ functional foods necessitates the development of products that are not only health-promoting but also possess high sensory quality. Fruit mousses are an excellent matrix for fortification, appealing to a wide consumer base. This study aimed to enrich a conventional apple–plum mousse with vitamin D_3_ and sea buckthorn oil, employing an encapsulation strategy based on endogenous fruit pectin. Three mousse variants were produced: a control (traditional), one fortified via the encapsulation of vitamin D_3_ and sea buckthorn oil in a pectin-based nanoemulsion, and one fortified via the direct addition of the bioactive compounds. The products were analysed using scanning electron microscopy (SEM), Fourier-transform infrared spectroscopy (FTIR), colorimetry, texture analysis, rheology, fatty acid profiling, and sensory evaluation (profiling and ranking). SEM and FTIR analyses confirmed the successful formation of spherical submicron capsules (approx. 100–300 nm) within the fortified mousse. Encapsulation resulted in significantly lower colour change (ΔE = 6.07 ± 0.03) compared to direct addition (ΔE = 11.16 ± 0.03). The fortified mousses exhibited approximately 16–20% lower hardness (0.21–0.22 N vs. 0.25 N) and threefold lower adhesiveness (0.06–0.08 N·s vs. 0.19 N·s) compared to the control. Rheological analysis indicated that fortification did not compromise the structural stability of the mousses (G′ > G″ across 0.1–10 Hz). The fatty acid profile was significantly improved in the fortified variants, with a three-fold increase in linoleic acid (C18:2 n-6 c: from 16.90% to 55–56%) and the introduction of γ-linolenic acid (C18:3 n-6: 0.38–0.39%). Sensory ranking revealed no significant differences in overall quality between the control and fortified mousses (*p* > 0.05). Pectin present in fruits can be effectively utilised to encapsulate vitamin D_3_ and sea buckthorn oil, allowing the successful development of a functional fruit mousse with an improved nutritional profile and retained sensory quality. Encapsulation proved to be a superior fortification method, offering better protection of bioactive compounds and a lesser impact on the product’s original colour.

## 1. Introduction

The contemporary food industry is undergoing a significant transformation driven by increasing consumer awareness. Modern consumers expect food that go beyond basic nutrition and provide measurable health benefits, stimulating the growth of the functional food market [1]. At the same time, the “clean label” trend promotes natural ingredients and minimal processing. Combining these two expectations—health-promoting and clean-label formulations—creates both a challenge and an opportunity for food manufacturers [2,3].

Fruit mousses and purees have evolved from traditional fruit preserves into convenient, ready-to-eat snacks, supported by advances in processing and packaging technology [4]. Their soft, homogeneous texture makes them particularly suitable for a wide range of consumers, including young children and the elderly, who may have difficulties with chewing and swallowing [5]. From a technological perspective, fruit purees are heterogeneous suspensions of cell wall particles dispersed in a water-rich serum containing dissolved sugars, phenolic compounds, and organic acids [6]. The texture, a key quality attribute, depends mainly on cell wall integrity and its degradation during processing [7]. Market data indicate a substantial increase in their consumption, with a significant percentage of both children and adults now consuming them regularly [4]. Recent research indicate that fruit-based purees enriched with bioactive compounds can achieve high consumer acceptance, confirming their potential as effective carriers for functional ingredients [5].

Apples (*Malus domestica* Borkh.) are among the most widely cultivated and consumed fruits worldwide and are a major raw material for fruit-based products. Their year-round availability, attractive taste, favourable nutritional profile make them a valuable component of functional foods [8,9]. Apples are a valuable source of bioactive compounds, including polyphenols, dietary fibre, vitamins, and triterpenic acids [10]. Notably, they are a significant dietary source of phenolic compounds, with approximately 22% of ingested polyphenols coming from this fruit [11]. These compounds, which have a high proportion of bioavailable free forms and are concentrated in the skin, are associated with a high antioxidant potential, contributing to the prevention of numerous chronic diseases, including cardiovascular diseases, diabetes, and certain cancers [9,12]. Furthermore, apples contain 10–15% pectin [13], a polysaccharide of both technological and thickening importance.

Pectin, a complex heteropolysaccharide and a soluble fraction of dietary fibre known for its numerous health-promoting benefits, including cholesterol reduction, and the regulation of glucose absorption [14]. In the context of food technology, and crucially for this work, pectin’s structure, which includes hydrophobic groups (acetyl and methoxyl), confers upon it emulsifying and stabilising capabilities. These properties make it an increasingly popular choice as a material for constructing encapsulation wall systems [15,16].

Plums (*Prunus domestica* and *P. salicina*) are widely cultivated stone fruits [17], valued for their taste and rich nutritional composition [18]. They contain bioactive compounds such as phenolic acids, vitamins, minerals, and dietary fibre [19]. They exhibit multi-directional effects on the human body, including supporting digestive health, cognitive function, and bone metabolism [20]. The concentration of polyphenols in the plum skin is about five times higher than in the pulp, justifying the use of whole fruit in mousse production [21].

The choice of fortificants—vitamin D_3_ and sea buckthorn oil—addresses two prevalent nutritional deficiencies. Vitamin D insufficiency is a global health problem, affecting an estimated one billion people worldwide [22]. This deficiency is pervasive across both developed and developing countries [23]. In Europe, a staggering 77–100% of adults were found to not consume sufficient vitamin D, leading to hypovitaminosis [24]. While its classical role in calcium and phosphorus metabolism is well-known, leading to deficiencies like rickets and osteoporosis, the discovery of vitamin D receptors in most human cells and tissues has expanded the understanding of its pleiotropic functions [25]. Deficiency is now linked to an increased risk of autoimmune thyroid diseases, gestational diabetes, respiratory infections, neurodegenerative diseases, certain cancers, and cardiovascular diseases [22,25,26]. Although the skin can synthesise vitamin D endogenously upon sun exposure, factors such as age, sunscreen use, geographical location, and season limit its efficacy [27]. Dietary sources, primarily fatty fish, liver, eggs, and cheese, are often insufficient to meet recommendations, necessitating strategies like food fortification [5,28].

Fortifying food with vitamin D presents a technological challenge due to the compound’s lipophilic nature, instability during processing, and susceptibility to degradation by oxygen and light during storage [23]. While direct addition is common, it can lead to increased degradation and interaction with packaging materials [5]. A more effective approach is encapsulation, which has been shown in numerous studies to successfully deliver vitamin D to various products, protecting it and ensuring high stability and bioavailability [23,29].

Sea buckthorn (*Hippophae rhamnoides*) oil, extracted from the fruits and seeds, is gaining popularity as a nutraceutical [30]. It contain rare palmitoleic acid, which support glucose metabolism and insulin secretion [31]. The seed oil is unique as a natural oil containing omega-3 and omega-6 fatty acids in an ideal 1:1 ratio [32]. Moreover, it is rich in carotenoids, tocopherols, and phytosterols, contributing to its strong antioxidant, anti-inflammatory, and hepatoprotective properties [31,33]. However, the incorporation of such oils into food systems is challenging due to the chemical instability of polyunsaturated fatty acids and their susceptibility to oxidation, as well as undesirable sensory attributes like a bitter or acidic taste [34]. Encapsulation offers an effective solution by protecting these labile compounds from degradation, masking unpleasant flavours, and enabling controlled release [35].

Encapsulation involves enclosing an active core material within a wall material [36]. It serves three key functions in the food industry: (a) protecting sensitive ingredients from environmental factors, (b) enabling controlled release of core materials, and (c) masking undesirable tastes or odours [35,37]. The choice of wall material is critical, and polysaccharides like pectin are increasingly favoured due to their natural origin, biocompatibility, non-toxicity, and beneficial structural properties [38]. Pectin can form various delivery systems, including nanoemulsions, hydrogels, and nano-/microcapsules, effectively increasing the stability and bioavailability of encapsulated nutraceuticals [14,16].

Despite the growing body of research on pectin-based encapsulation systems, there remains a significant gap in the literature regarding the utilisation of endogenous fruit pectin—naturally present in the food matrix itself—as an in situ encapsulating agent. Most studies employ commercially extracted or purified pectin added as an external ingredient. The novelty of this approach lies in exploiting the inherent pectin content of the fruit puree to simultaneously serve both as a structural component and as a natural wall material for bioactive delivery, thereby achieving true ‘clean label’ fortification without additional encapsulation agents. Furthermore, the co-encapsulation of both fat-soluble vitamin D_3_ and omega-rich sea buckthorn oil within a single pectin-based system, delivered through a widely consumed fruit-based product, represents an innovative strategy for addressing multiple nutritional deficiencies simultaneously.

Therefore, this study aims to address the growing demand for “clean label” functional foods by developing a fortified apple–plum mousse. The innovation lies in utilising the endogenous pectin from the fruits themselves as a natural encapsulating material for the simultaneous delivery of vitamin D_3_ and sea buckthorn oil. The objective is to enhance the nutritional profile of a conventional product by introducing essential fatty acids and addressing a widespread micronutrient deficiency, while employing an encapsulation strategy designed to minimise the impact on the product’s sensory qualities and maximise the stability of the bioactive compounds. The physicochemical and sensory properties of the resulting fortified mousses was comprehensively evaluated and compared to a traditional control and a mousse fortified by direct addition.

## 2. Results and Discussion

### 2.1. Visual Assessment of Fruit Mousses

**Figure 1:**
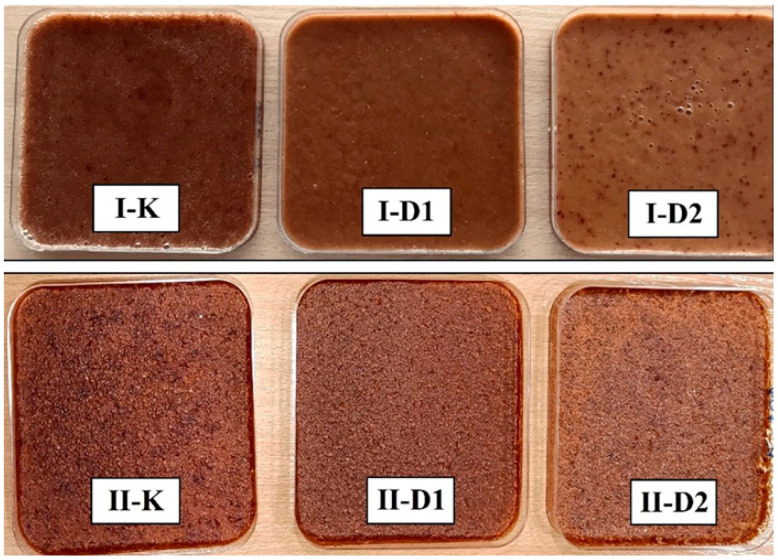
Visual appearance of fresh (**top row**) and dried fruit mousses (**bottom row**): control (K), mousse fortified with vitamin D_3_ via nanoemulsion encapsulation (D1), and mousse with direct vitamin D_3_ addition (D2).

A visual comparison of the freshly prepared and dried mousses is presented in Figure 1. All variants exhibited a characteristic brownish hue derived from the fruit raw materials. However, distinct differences in colour intensity and texture were observed. The mousses fortified with vitamin D_3_ and sea buckthorn oil (D1 and D2) appeared visibly lighter than the traditional control mousse (K). The colour change was less pronounced in the mousse fortified via encapsulation (D1) compared to the one with direct addition (D2). This can be attributed to the reduction in oil droplet size during nanoemulsion formation via ultrasonication [9] and the subsequent encapsulation within a pectin matrix, which isolates the oil and its pigments from the external environment, leading to higher homogeneity and a lesser impact on the product’s colour [14]. Furthermore, the dried sample of the encapsulated mousse (D1) exhibited a more uniform, homogeneous, and smoother texture compared to the other variants.

### 2.2. Physicochemical Properties of Apple–Plum Mousses

#### 2.2.1. Morphology of the Fortified Mousse via Encapsulation

**Figure 2:**
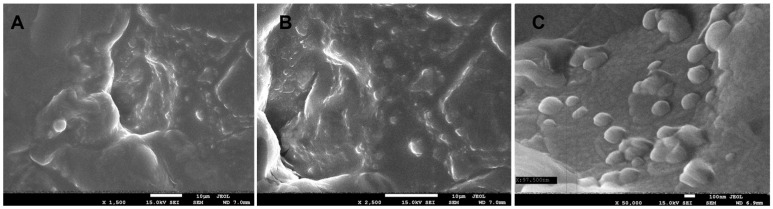
Scanning Electron Microscopy (SEM) micrographs of the dried encapsulated mousse (D1) at different magnifications: (**A**) 1500×, (**B**) 2500×, (**C**) 50,000×.

Scanning Electron Microscopy (SEM) was employed to visualise the morphology of the dried encapsulated mousse (D1). The images (Figure 2) revealed a heterogeneous surface with the presence of spherical structures dispersed throughout the food matrix. These structures, with a size of approximately 100–300 nm, are indicative of successfully formed submicron capsules. This confirms that the core material (vitamin D_3_ dissolved in sea buckthorn oil) was encapsulated by the wall material, primarily comprising pectin and other polysaccharides from the fruit raw materials. Although the encapsulation efficiency (EE%) was not directly quantified in this study, the SEM observations confirm successful capsule formation. Based on comparable pectin-based encapsulation systems reported in the literature, EE values typically range from 70% to 95% for lipophilic bioactives when using ultrasonic emulsification followed by pectin wall formation [39,40]. The high retention of fatty acids observed in sample D1 compared to D2 (Section 2.2.6) indirectly suggests effective encapsulation and protection of the oil phase.

#### 2.2.2. Structure of the Products

**Figure 3:**
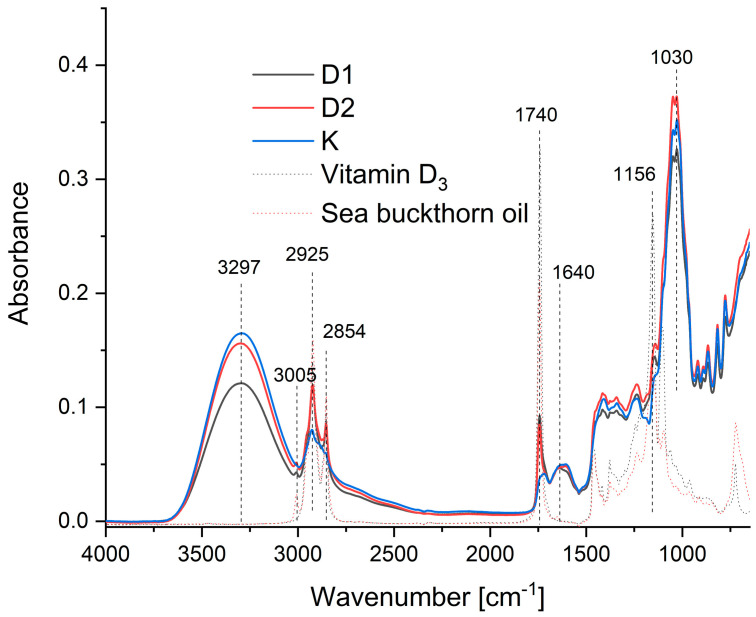
FTIR spectra of the analysed samples: control mousse (K), mousse fortified via encapsulation (D1), mousse fortified via direct addition (D2), pure vitamin D_3_, and pure sea buckthorn oil.

FTIR spectroscopy was used to identify structural differences between the mousse variants (Figure 3). All fruit mousses showed a broad absorption band at around 3300 cm^−1^, characteristic of O-H stretching vibrations, which can be attributed to pectin and water [41]. The intensity of this band was lowest in the encapsulated mousse (D1), suggesting that the hydroxyl groups of pectin were utilised in forming and stabilising the spherical capsule structures, thereby reducing the free O-H signal.

In the spectra of the fortified mousses, a slight increase in intensity was observed at 3000 cm^−1^, a region associated with cis-olefinic =C-H stretching vibrations of the oil [42]. This signal was less intense in the encapsulated mousse than in the direct addition variant, supporting the notion that encapsulation can mask the core components [37]. Peaks at ~2850 cm^−1^ (CH_2_ stretching) and ~1750 cm^−1^ (C=O stretching), characteristic of both the oil and vitamin D_3_ [43,44], appeared in the fortified mousses, confirming the presence of the enriching compounds. The intensity of the signal at ~1640 cm^−1^ (free carboxyl groups of pectin) was identical for all mousses, indicating these groups were not involved in bonding with the fortificants. These results collectively confirm the successful incorporation of the bioactive compounds and the structural role of pectin in encapsulation.

At the molecular level, the encapsulation mechanism can be attributed to pectin’s amphiphilic character. Pectin molecules possess hydrophobic groups (acetyl and methoxyl esters) along with hydrophilic carboxyl and hydroxyl groups. During ultrasonic emulsification, the mechanical energy reduces oil droplet size to the submicron range, creating a large oil–water interfacial area. Pectin molecules adsorb at this interface with their hydrophobic moieties oriented toward the oil phase (containing vitamin D_3_ and sea buckthorn oil) and hydrophilic groups extending into the aqueous phase. Electrostatic repulsion between negatively charged carboxyl groups (at the pH of the fruit matrix, typically 3–4) provides primary stabilisation, while hydrogen bonding between hydroxyl groups facilitates network formation around the droplets. This creates a protective layer that sterically and electrostatically stabilises the emulsion droplets, eventually forming the observed capsule wall structures [45,46,47]. The reduced intensity of the O-H stretching band at 3300 cm^−1^ in sample D1 (Figure 3) supports this hypothesis, indicating that hydroxyl groups are engaged in intermolecular interactions within the capsule structure rather than remaining free.

#### 2.2.3. Colour of the Products

Instrumental colour measurements (Table 1) revealed statistically significant differences (*p* < 0.05) between the mousse variants for all parameters (L*, a*, b*). The parameter L* (lightness) increased in the following order: traditional mousse < encapsulated mousse (D1) < direct addition mousse (D2). This aligns with the visual assessment and is a direct result of introducing the orange-coloured sea buckthorn oil [48], which lightened the original brown colour of the fruits. Similar lightening effects have been observed in other desserts fortified with nanoemulsions containing oil [5]. The observed brightness and chromatic changes can also be explained by optical phenomena related to the size and distribution of droplets. In emulsified systems, smaller and more equally dispersed droplets increase light scattering, improving brightness (higher L* value). Encapsulation through the creation of nano- or micro-sized structures clearly affects light scattering in the product [49]. According to McClements [49] the observed increase in emulsion lightness can be attributed to more intensive multiple scattering of light by the dispersed oil droplets, which enhances overall reflectance. From a sensory standpoint, higher droplet concentra-tions result in a lighter and less saturated colour appearance of the emulsions.

All mousses exhibited positive a* (redness) and b* (yellowness) values. The higher a* and b* values in the fortified mousses are attributed to the high concentration of yellow-orange carotenoids in sea buckthorn oil [48]. The encapsulated mousse (D1) showed significantly lower a* and b* values than the direct addition mousse (D2). This could be due to the colour-masking effect of the submicron capsules themselves [35]. Sample D2 exhibited the highest colour saturation (C > 26). The dark orange hue of the products results from the presence of various natural pigments and phenolic compounds inherent in the raw materials used. In this sample, pigments derived from propolis and sea buckthorn oil predominate. The lower colour saturation observed in sample D1 indicates that the encapsulation process partially masks the colour intensity of the encapsulated compounds. The lower h* value observed for sample D2 (0.79 ± 0.00) compared to D1 (0.89 ± 0.00) confirms a shift in hue toward reddish–orange tones, likely resulting from the presence of natural pigments from propolis and sea buckthorn oil. The total colour difference (Δ*E*) between the control sample and the fortified mousses confirmed the clear visual changes. Sample D1 showed a noticeable difference (Δ*E* = 6.07 ± 0.03), while sample D2 showed a very strong deviation (Δ*E* = 11.16 ± 0.03). According to commonly accepted thresholds (Δ*E* > 3—visually noticeable; Δ*E* > 5—clear difference), these results indicate that both the additives in capsules and without capsules had a significant effect on the colour of the product, with the effect being more pronounced in the case of the preparation without capsules. The observed differences indicate that oils containing natural pigments can be enclosed within submicron capsules, whose pectin-based walls effectively isolate them from the external environment. As a result, the pigments do not scatter light throughout the entire volume of the mousse, and their colour does not dominate over the natural hue of the fruit matrix [50,51]. Encapsulation can therefore mask the colour of added ingredients by limiting the diffusion of pigments into the matrix and altering the way light is scattered and reflected within the product. As a result, a mousse containing encapsulated additives exhibits a more natural, uniform, and less intense colour compared to a mousse with non-encapsulated additions.

#### 2.2.4. Texture of the Products

The textural parameters of the mousses are summarised in Table 2. The hardness of all mousses was relatively low (0.21–0.25 N), which is consistent with the expected mechanical disruption of the fruit cell wall matrix occurring during homogenisation [7]. The fortified mousses (D1 and D2) were significantly softer and displayed approximately threefold lower adhesiveness compared to the control. Such behaviour can be attributed to the incorporation of plant-derived lipids, which enhance lubricity and creaminess, thereby softening the structure and reducing adhesive forces [11]. Oil, as a hydrophobic substance, forms a thin layer on the surface of the product, which limits direct contact between the product and the measuring surface, such as the texture analyser probe or the consumer’s tongue. By reducing friction and cohesive forces between the particles and the surface, the force required to detach the sample is decreased. These changes may result from the disruption of the gel network formed by polysaccharides (e.g., pectin, starch) or from a reduced ability to form hydrogen bonds between the sample and the surface. From a sensory perspective, consumers perceive a decrease in viscosity and stickiness of the product to the palate or spoon, leading to a smoother and creamier mouthfeel [52,53,54].

#### 2.2.5. Rheological Properties of the Products

**Figure 4:**
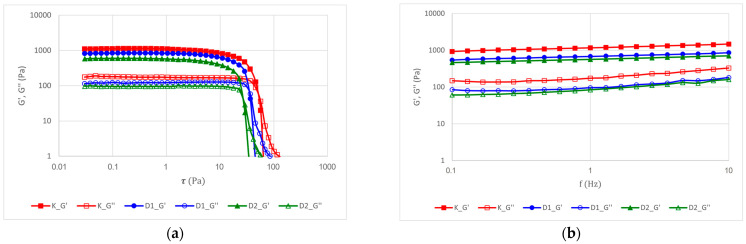
Rheological Properties of the Products: curves of the storage (G′) and loss (G″) moduli of fruit mousses (**a**) and curves of the storage (G′) and loss (G″) moduli of fruit mousses as a function of frequency (**b**).

The rheological behaviour of the mousses was characterised by strain and frequency sweep tests. In the strain sweep (Figure 4a), all mousses exhibited a linear viscoelastic region (LVR) at low strains (<10 Pa), where the storage (G′) and loss (G″) moduli were constant, indicating a stable structure. Beyond a critical strain of 10 Pa, a sharp decrease in both moduli was observed, signifying the irreversible breakdown of the mousse structure [8]. The addition of sea buckthorn oil and propolis caused a slight decrease in the structural stability of the mousses, which was more evident in the sample containing non-encapsulated additives (D2). This finding is consistent with the observations of Wang et al. [55]. Samples with higher oil concentrations exhibited a more pronounced decrease in storage modulus compared to those with lower oil content, indicating that increased oil addition leads to a less stable gel structure under large deformation.

The encapsulation process partially mitigated the adverse effect of lipids and propolis, suggesting improved structural integrity resulting from the controlled release of the oil phase. The storage modulus (G′) was higher than the loss modulus (G″) across the entire frequency range tested (0.1–10 Hz, Figure 4b), confirming the solid-like, gel behaviour of the fruit purees [56]. The fortified mousses (D1 and D2) showed slightly lower values of both moduli compared to the control. This indicates that the addition of the oil did not compromise the structural stability of the mousses, which can be attributed to the stabilising role of pectin at the oil–water interface through steric and electrostatic interactions [15,57]. Encapsulation diminishes the lubrication effect of the oil, allowing for controlled modulation of the textural and rheological properties of the fruit mousses. The type and composition of the polymer used to form the capsule shell significantly affect the encapsulation efficiency and the release behaviour of the encapsulated compounds [36,58]. Previous studies have demonstrated that pectin-based encapsulation systems significantly enhance the stability of lipophilic vitamins and polyunsaturated fatty acids during storage [39]. The protective effect observed in our fatty acid analysis (Table 3), where encapsulated samples showed higher retention of most fatty acids compared to direct addition, suggests that the pectin-based system may similarly protect vitamin D_3_ and limit PUFA oxidation over time. This finding is particularly relevant, as the sensory perception of consistency in food emulsions is closely associated with their rheological behaviour. Numerous studies have demonstrated that parameters such as oil volume fraction, droplet size, and emulsion viscosity directly influence lubrication properties and the perception of fat-related sensory attributes in such systems [52].

#### 2.2.6. Fatty Acid Profile

The fatty acid composition was significantly altered by fortification (Table 3). The fortified mousses contained short- and medium-chain fatty acids (C4:0, C8:0, C10:0, C10:1) and γ-linolenic acid (C18:3 n-6), which were absent in the control. Most notably, the content of the essential omega-6 linoleic acid (C18:2 n-6 c) increased approximately threefold, consistent with the known profile of sea buckthorn seed oil [12]. The encapsulated mousse (D1) showed significantly higher levels of most fatty acids compared to the direct addition mousse (D2), with the exception of C16:1, C18:1 n7, and C18:2 n-6 c. This suggests that the encapsulation technology provided better protection against the oxidation of polyunsaturated fatty acids during processing, as demonstrated in other studies where microencapsulation improved the oxidative stability of oils rich in PUFAs [12,59].

**Table 3:** Fatty acid profile (% of total fatty acids) of the apple–plum mousses (mean ± SD). Values in the same row marked with different letters (a, b, c) differ statistically significantly (*p*, 0.05). The letters indicate membership in statistically homogeneous groups determined by Tukey’s test and do not indicate a ranking of values from highest to lowest.

Fatty Acids [%]		Product	
	K	D1	D2
C4:0	0.00 ± 0.00 ^a^	0.69 ± 0.03 ^b^	0.58 ± 0.01 ^c^
C8:0	0.00 ± 0.00 ^a^	0.78 ± 0.04 ^b^	0.60 ± 0.10 ^c^
C10:0	0.00 ± 0.00 ^a^	0.62 ± 0.02 ^b^	0.55 ± 0.00 ^c^
C10:1	0.00 ± 0.00 ^a^	0.18 ± 0.00 ^b^	0.14 ± 0.00 ^c^
C14:0	1.61 ± 0.01 ^a^	0.00 ± 0.00 ^b^	0.00 ± 0.00 ^b^
C16:0	27.65 ± 0.03 ^a^	8.45 ± 0.03 ^b^	8.22 ± 0.02 ^c^
C16:1	1.75 ± 0.03 ^a^	0.75 ± 0.00 ^b^	0.76 ± 0.01 ^b^
C18:0	13.42 ± 0.00 ^a^	5.52 ± 0.02 ^b^	5.15 ± 0.00 ^c^
C18:1 n-9 c	33.21 ± 0.02 ^a^	25.23 ± 0.03 ^b^	24.98 ± 0.01 ^c^
C18:1 n7	2.66 ± 0.00 ^a^	1.08 ± 0.01 ^b^	1.09 ± 0.01 ^b^
C18:2 n-6 c	16.90 ± 0.01 ^a^	55.13 ± 0.01 ^b^	56.39 ± 0.06 ^c^
C18:3 n-6	0.00 ± 0.00 ^a^	0.39 ± 0.01 ^b^	0.38 ± 0.00 ^b^
C18:3 n-3	2.81 ± 0.02 ^a^	1.18 ± 0.00 ^b^	1.17 ± 0.02 ^b^

### 2.3. Sensory Analysis of Fruit Mousses

#### 2.3.1. Sensory Quality of the Products

**Figure 5:**
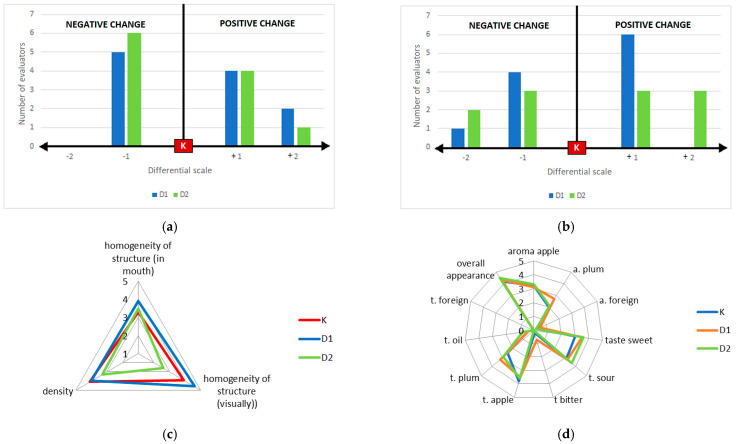
(**a**) Differential profile regarding the surface appearance of fruit mousses; (**b**) Differential profile regarding the colour of fruit mousses; (**c**) Profile analysis results of texture; (**d**) Profile analysis results of aroma, taste and overall appearance.

The surface appearance of the traditional mousse (K) was considered the standard, reflecting the typical look of a fruit mousse, which exhibits no syneresis, no separation of ingredients, no visible fat phase, and has a smooth, glossy, but not shiny structure; the presence of small fruit skin particles was, however, acceptable. The product modification introduced an oil phase into the mousse, and the panellists observed changes in the appearance of both fortified mousses. The observation of bubbles and/or oil droplets was considered a negative attribute, whereas complete product homogenisation was positive. Regarding colour, it was characteristic of the fruits from which the mousses was produced, and in this case, the mousse (K) was deemed the reference product. The panellists’ assessment of the external appearance indicated that the fortified samples differed from the control. However, differential profiling of external appearance showed no clear consensus among panellists on whether the changes in the surface appearance and colour of the fortified mousses was positive or negative. A key finding was that the encapsulated mousse (D1) was perceived as having a less intensely changed colour compared to the mousse with direct addition (D2), with 10 out of 11 panellists rating its colour change as ‘slightly changed’. The results of the sensory analysis for this attribute are consistent with the instrumental colour assessment presented in Table 2 (ΔE value) and reflect the results of the colour parameter analysis, which confirm that the fortified mousses differ in colour from the control mousse (Figure 5).

Regarding consistency, the fortified mousses was assessed as more homogeneous than the control. The encapsulated mousse (D1) received the highest score for homogeneity in the mouth. Fortified mousses were also perceived as less dense, consistent with the instrumental texture analysis.

The flavour profile (Figure 5d) revealed that the fortified mousses had a more intense plum flavour and were perceived as sweeter and more acidic than the control. The encapsulated mousse (D1) had a lower perceived acidity than the direct addition variant (D2), suggesting partial masking of the sour notes from the sea buckthorn oil, a known benefit of encapsulation [35]. A slight aftertaste (described as oily or astringent) was noted by a few panellists for both fortified mousses, with no significant difference between the methods. Crucially, the overall appearance (Figure 5d) for all mousses was above 4.0 on a 5-point scale, and no differences were found between the control and the fortified variants. This demonstrates that the enrichment did not compromise the overall sensory acceptability of the product.

#### 2.3.2. Sensory Quality Assessment by Ranking

The sensory quality of the mousses was assessed using the ranking method. The highest sensory quality of a product was equivalent to assigning it to 1st place. The rank refers to the position assigned to the evaluated product by a panellist (1st place—rank 1, 2nd place—rank 2, 3rd place—rank 3). The summed ranks are presented in Table 4.

The evaluated apple–plum mousses did not differ significantly in terms of sensory quality. It was demonstrated that modifying conventional products to enhance health benefits does not necessarily lead to a reduction in the sensory quality of the product.

## 3. Materials and Methods

### 3.1. Materials

Apples (*Malus domestica*) and plums (*Prunus domestica*), comprising two varieties, were procured from a local market. For the production of the fortified mousses, a commercial preparation of vitamin D_3_ (20,000 IU/mL, Lusomedicamenta Sociedade Técnica Farmacêutica, S.A., Barcarena, Portugal) and cold-pressed sea buckthorn oil (LLC LekAltai Barnaul, Altai Krai, Russia) were used as fortificants. All chemicals and solvents used for analyses were of analytical grade.

### 3.2. Preparation of Fruit Mousses

Three distinct variants of apple–plum mousses were manufactured, as outlined in Table 5.

#### 3.2.1. Traditional Fruit Mousse

Apples were cored, and plums were pitted. The prepared fruits were combined in a 1:1 ratio to a total mass of 1000 g. Subsequently, 100 mL of distilled water was added, and the mixture was homogenised using a kitchen blender (Götze & Jensen, Copenhagen, Denmark) until a homogeneous puree was achieved. The final yield was ten 100 mL portions.

#### 3.2.2. Fortified Mousse via Encapsulation

The base mousse was prepared as described in Section 3.2.1. The encapsulation process involved the creation of a nanoemulsion: 10 drops (equivalent to 0.25 mL) of vitamin D_3_ were dissolved in 10 g of sea buckthorn oil, and 10 g of water was added. This mixture was then homogenised using an ultrasonic homogeniser (Bandelin electronic GmbH & Co. KG, Berlin, Germany) at 40% amplitude (corresponding to approximately 20 W output power) for 5 min in continuous mode at room temperature (20–22 °C) to reduce the oil droplet size and form a nanoemulsion. The resulting nanoemulsion was introduced dropwise via an automatic pipette into a beaker containing a portion of the mousse under constant agitation using a high-speed homogeniser (Polytron PT 2500 E, Kinematica AG, Luzern, Switzerland) at 13,300 rpm to ensure even distribution. This mixture was then incorporated into the remaining mousse volume and mixed thoroughly. Each 100 mL portion of the final product contained 12.5 µg of cholecalciferol (500 IU of vitamin D_3_) and 1 g of sea buckthorn oil.

#### 3.2.3. Fortified Mousse via Direct Addition

The base mousse was prepared as described in Section 3.2.1. Following the homogenisation step, 10 g of water, 10 g of sea buckthorn oil, and 10 drops vitamin D_3_ were added directly to the mousse and mixed thoroughly to ensure uniform dispersion. The final product yield was ten 100 g portions.

### 3.3. Physicochemical Analysis

#### 3.3.1. Sample Preparation

For subsequent analyses, 70 g of each mousse variant (Control, D1, D2) were dried at 40 °C for approximately 24 h. The dried samples were utilised for Scanning Electron Microscopy (SEM), Fourier-Transform Infrared Spectroscopy (FTIR), and fatty acid analysis. Colour, texture, rheological measurements, and sensory evaluation were performed on freshly prepared mousses.

#### 3.3.2. Scanning Electron Microscopy (SEM)

The morphology of the dried encapsulated mousse (D1) was examined using a JEOL 7550 scanning electron microscope (JEOL Ltd., Akishima, Tokyo, Japan). To enhance conductivity, the sample was sputter-coated with a 20 nm layer of chromium using a K575X Turbo Sputter Coater (Emitech Ltd., Kent, UK).

#### 3.3.3. Fourier-Transform Infrared Spectroscopy (FTIR)

FTIR spectra were acquired for all dried mousse variants, as well as for the pure vitamin D_3_ preparation and sea buckthorn oil. A MATTSON 3000 FT-IR spectrophotometer (Madison, WI, USA) equipped with a MIRacle ATR accessory (PIKE Technologies Inc., Madison, WI, USA) was employed. Spectra were collected at 20 °C (±2 °C) across a wavenumber range of 4000–700 cm^−1^.

#### 3.3.4. Colour Measurement

Instrumental colour analysis was performed according to a previously established methodology [61]. A Konica Minolta CM-3500d spectrophotometer (Konica Minolta Inc., Tokyo, Japan) with a 30 mm measurement window was used. The measurements were taken using a D65 illuminant and a 10° standard observer. The CIE L*a*b* colour space was used, where L* represents lightness (0 = black, 100 = white), a* indicates the green (−a) to red (+a) axis, and b* indicates the blue (−b) to yellow (+b) axis. The measurements were carried out in five repetitions using a white background as a reference.

Furthermore, the colour parameters C* and h* were calculated. Chroma (C*), also referred to as saturation, represents how vivid or intense a colour appears in comparison to a neutral grey of equal lightness. Higher C* values indicate a greater colour intensity as perceived by the human eye. This parameter was computed according to the following equation [62]:

$$C^{*}=\;\sqrt{{a^{*}}^{2}+{b^{*}}^{2}}$$

The hue angle (h*) represents the position of a colour on a three-dimensional colour wheel, where 0° corresponds to red, 90° to yellow, 180° to green, and 270° to blue, as perceived by the human eye [63]. It was determined using the following formula:

$$h^{*}={tan}^{-1}\left(\frac{b^{*}}{a^{*}}\right)$$

#### 3.3.5. Texture Analysis

Textural properties of the samples were evaluated using a TA.XTplus texture analyser (Stable Micro Systems Ltd., Godalming, UK). A penetration test was performed using a cylindrical probe with a diameter 20 mm. The probe penetrated the sample, placed in a container of 55 mm diameter jar, to a depth of 25 mm at a speed of 1 mm/s, with a trigger force of 5 g. From the obtained force-time curves, hardness (maximum force of the first compression, expressed in Newtons, N) and adhesiveness (the negative area under the curve, corresponding to the work required to detach the probe from the sample, expressed in Newton-seconds, N·s) were determined. All measurements were performed on samples equilibrated to 8 ± 1 °C. All measurements were performed in triplicate.

#### 3.3.6. Rheological Measurements

The rheological properties of freshly prepared mousses were analysed using a RheoStress RS6000 rotational rheometer (Thermo Scientific, Karlsruhe, Germany) equipped with a P35Ti plate geometry. The temperature of the plate was maintained at 25.0 ± 0.1 °C. Samples were equilibrated at 25 °C for 1 h prior to analysis. All measurements were performed in triplicate.

Strain sweep tests: The test was performed to identify the linear viscoelastic region (LVR) by increasing the oscillatory stress logarithmically from 0.1 to 100 Pa at a constant frequency of 1 Hz.

Frequency sweep tests: The test was conducted within the LVR at a constant strain of 0.1 Pa, varying the oscillation frequency from 0.1 to 10 Hz.

For both experiments, the storage modulus (G′) and loss modulus (G″) were recorded as functions of strain and frequency, respectively, providing insights into the elastic and viscous characteristics of the mousse systems.

#### 3.3.7. Fatty Acid Analysis

The fatty acid profile was determined based on the methodology described by Turek et al. [8]. Briefly, lipids were extracted from the dried samples using a cold extraction method (modified Folch method) with a chloroform-methanol mixture (2:1, *v*/*v*). The extracted lipids were then transesterified to form fatty acid methyl esters (FAMEs).

The FAME analysis was conducted using a Thermo Electron Corporation TRACE GC ULTRA gas chromatograph (Thermo Fisher Scientific, Waltham, MA, USA) equipped with a flame ionisation detector (FID) and a BPX-70 capillary column (60 m × 0.20 mm, 0.25 µm film thickness). The temperature programme was as follows: initial temperature 60 °C (held for 3 min), increased at 7 °C/min to 200 °C (held for 20 min). The injector and detector temperatures were set at 220 °C. A split injection mode (10:1) was used with helium as the carrier gas at a flow rate of 5 mL/min. Peaks were identified by comparing their retention times with those of a Supelco 37 FAME Mix standard (Sigma-Aldrich Co., St. Louis, MO, USA). Each sample was analysed in duplicate, with two injections per replicate.

### 3.4. Sensory Analysis

The sensory evaluation was conducted by a trained panel of 11 assessors, selected and trained in accordance with the standards [64,65,66,67,68,69]. All tests were performed in a sensory laboratory designed to meet the requirements of PN-EN ISO 8589:2007 [70]. The study protocol was approved by the University Ethics Committee for Research Involving Humans (approval number 274/2025). The methods employed included sensory profiling [68,71] and ranking [60]. The assessors (panellists) evaluated three samples of mousses (K, D1, D2), which were served in odourless, transparent plastic cups, each containing a 30 g portion, and coded with random three-digit numbers. All samples were assessed at room temperature. The samples were prepared one day in advance and stored in a refrigerator at 4 °C.

#### 3.4.1. Sensory Profiling

Sensory profiling (QDA—Quantitative Descriptive Analysis) was carried out based on the standard PN-EN ISO 13299:2016 [68]. The panel evaluated the following descriptors across four attribute groups:

(a) External Appearance: surface appearance, colour.
(b) Consistency: homogeneity of consistency (visually), homogeneity of consistency (in the mouth), density.
(c) Smell (Odour): apple, plum, foreign.
(d) Taste: sweet, sour, bitter, apple, plum, oily, and foreign taste.

An overall product assessment was also performed.

For the evaluation of External Appearance and Consistency, a variant of the QDA method known as differential profiling was used, whereas for the remaining attributes, the classic QDA description was applied.

For the evaluation of external appearance, a differential profiling method was used, with the traditional mousse (Control-K) serving as the reference standard (“S”). A two-directional scale was employed, where deviation to the left (“−”) indicated a negative/unfavourable change from the standard, and deviation to the right (“+”) indicated a positive/favourable change. The intensity of the difference was rated as 1 (“slightly changed”) or 2 (“very changed”). For consistency, smell (odour), taste, and overall quality, a 5-point structured scale with boundary definitions was used (Table 6). The evaluation form used is provided in the Appendix A.

#### 3.4.2. Ranking Test

The ranking test was performed according to the standard ISO 8587:2006 [60]. Assessors were presented with three coded samples of the fruit mousses simultaneously. They were instructed to taste the samples in a balanced order and to rank them according to their overall preference, from the most preferred (rank 1) to the least preferred (rank 3). The Friedman test was applied to the rank sums at a significance level of α = 0.05 to identify significant differences between samples.

#### 3.4.3. Statistical Analysis

For the physicochemical analysis, statistical evaluation was performed using Statistica software, version 13.3 (StatSoft, Tulsa, OK, USA). One-way analysis of variance (ANOVA) and Fisher’s test were applied (*p* < 0.05). To analyse the results of the sensory quality assessment of apple and plum mousses obtained using the ranking method, the Friedman test was applied (α = 0.05). Tukey’s test was applied to analyse the results of the fatty acid profile (*p* < 0.05). All results are presented as mean ± standard deviation.

## 4. Conclusions

This study successfully demonstrated the development of a ‘clean label’ functional apple–plum mousse enriched with vitamin D_3_ and sea buckthorn oil using endogenous fruit pectin as a natural encapsulation material. SEM confirmed the formation of spherical submicron capsules (~100–300 nm), while FTIR spectroscopy validated the structural role of pectin in the encapsulation process.

Encapsulation proved superior to direct addition in multiple aspects: (1) significantly lower colour impact (ΔE = 6.07 vs. 11.16), preserving the product’s visual appeal; (2) enhanced protection of polyunsaturated fatty acids, with higher retention of most fatty acids; and (3) improved texture properties, with reduced hardness (0.21 N vs. 0.25 N) and adhesiveness (0.06 N·s vs. 0.19 N·s) that may benefit specific consumer groups such as the elderly. Rheological stability (G′ > G″ across all frequencies) was maintained in all variants.

Nutritional enhancement was substantial: linoleic acid content increased three-fold (from 16.90% to 55–56%), and γ-linolenic acid was successfully introduced (0.38–0.39%). Critically, sensory evaluation revealed no significant differences in overall quality among samples, confirming consumer acceptability.

From an industrial perspective, the proposed encapsulation method demonstrates promising scalability potential. The process utilises readily available equipment (ultrasonic homogenizers and high-speed mixers) commonly found in food processing facilities. Ultrasonic processing at industrial scale has been successfully implemented in various food applications, including emulsion production and extraction processes. The use of endogenous fruit pectin eliminates the need for additional encapsulation materials, reducing costs and maintaining the clean label character of the product. Energy requirements for ultrasonic emulsification are moderate compared to high-pressure homogenization, and the process can be integrated into existing fruit processing lines.

In summary, endogenous pectin-based encapsulation offers a natural, label-friendly solution for stabilising and delivering sensitive lipophilic compounds in fruit products. This strategy enhances nutritional value while preserving sensory and visual quality, supporting the development of innovative clean-label functional foods.

## Figures and Tables

**Table 1:** Colour parameters of the different apple–plum mousse variants.

Sample	L* (D65)	a* (D65)	b* (D65)	C*	h* (rad)	Δ*E*
K	35.01 ± 0.04 ^a^	12.32 ± 0.06 ^c^	12.12 ± 0.04 ^c^	17.29 ± 0.05 ^c^	0.78 ± 0.00 ^c^	
D1	38.23 ± 0.01 ^b^	13.89 ± 0.03 ^b^	17.06 ± 0.03 ^b^	22.00 ± 0.04 ^b^	0.89 ± 0.00 ^a^	6.07 ± 0.03
D2	41.29 ± 0.03 ^c^	18.68 ± 0.01 ^a^	18.85 ± 0.03 ^a^	26.54 ± 0.03 ^a^	0.79 ± 0.00 ^b^	11.16 ± 0.03

The values are expressed as the mean ± standard deviation. The presence of the same superscript letter (a, b, c) in each column indicates that there is no statistically significant difference between the values (*p* < 0.05).

**Table 2:** Texture parameters of the different apple–plum mousse variants.

Sample	Texture Parameters	
	Hardness [N]	Adhesiveness [N·s]
K	0.25 ± 0.01 ^a^	0.19 ± 0.03 ^a^
D1	0.21 ± 0.00 ^c^	0.06 ± 0.00 ^b^
D2	0.22 ± 0.00 ^b^	0.08 ± 0.00 ^b^

The values are expressed as the mean ± standard deviation. The presence of the same superscript letter (a, b, c) in each column indicates that there is no statistically significant difference between the values (*p* < 0.05).

**Table 4:** Sensory quality assessment of apple–plum mousses by the ranking method.

Product	Sum of Ranks
K	24 ^a^
D1	21 ^a^
D2	21 ^a^

Superscript letters denote homogenous groups (Friedman test, α = 0.05) [ISO 8587:2006] [60].

**Table 5:** Formulation of the prepared fruit mousse variants.

Sample Designation	Mousse Variant Description
Control (K)	Traditional apple–plum mousse
Fortified 1 (D1)	Apple–plum mousse enriched with vitamin D_3_ and sea buckthorn oil via encapsulation
Fortified 2 (D2)	Apple–plum mousse enriched with vitamin D_3_ and sea buckthorn oil via direct addition

**Table 6:** Definitions of selected attributes.

Attribute	Feature
External Appearance	Presence/Absence of: syneresis (water leakage), ingredient separation, visible oil droplets; surface shine/gloss. Colour
Consistency	Homogeneity of consistency (visually), homogeneity of consistency (in the mouth), density
Smell (Odour)	apple, plum, foreign
Taste	Sweet, sour, bitter, apple, plum, oily, and foreign taste.

## Data Availability

The data presented in this study are available on request from the corresponding author. The data are not publicly available due to privacy restrictions and ongoing research utilizing the same dataset.

## Abbreviations

The following abbreviations are used in this manuscript:

SEM	Scanning Electron Microscopy
FTIR	Fourier-Transform Infrared Spectroscopy
PUFA	Polyunsaturated Fatty Acids
QDA	Quantitative Descriptive Analysis
FAME	Fatty Acid Methyl Esters
GC-FID	Gas Chromatography with Flame Ionisation Detector
ATR	Attenuated Total Reflectance
CIE Lab*	Commission Internationale de l’Éclairage Lightness and Color Coordinates
ISO	International Organization for Standardization
PN-EN	Polish Standard—European Norm

## Appendix A. Definitions of Descriptors Selected

□	□	K		□	□		
−2	−1			+1	+2		
unfavourable features			favourable features				
							

	1	2	3	4	5		
							
inhomogeneity				very homogeneity			
	0	1	2	3	4	5	
							
undetactable				strongly detactable			
							
	0	1	2	3	4	5	
							
undetactable				strongly detactable			
